# Aging of *Podospora anserina* Leads to Alterations of OXPHOS and the Induction of Non-Mitochondrial Salvage Pathways

**DOI:** 10.3390/cells10123319

**Published:** 2021-11-26

**Authors:** Verena Warnsmann, Jana Meisterknecht, Ilka Wittig, Heinz D. Osiewacz

**Affiliations:** 1 Institute of Molecular Biosciences, Faculty of Biosciences, Goethe-University, Max-von-Laue-Str. 9, 60438 Frankfurt, Germany; warnsmann@bio.uni-frankfurt.de; 2 Functional Proteomics, Institute of Cardiovascular Physiology, Faculty of Medicine, Goethe-University, Theodor-Stein-Kai 7, 60590 Frankfurt am Main, Germany; meisterknecht@em.uni-frankfurt.de (J.M.); wittig@med.uni-frankfurt.de (I.W.)

**Keywords:** *Podospora anserina*, aging, OXPHOS, complexome profiling

## Abstract

The accumulation of functionally impaired mitochondria is a key event in aging. Previous works with the fungal aging model *Podospora anserina* demonstrated pronounced age-dependent changes of mitochondrial morphology and ultrastructure, as well as alterations of transcript and protein levels, including individual proteins of the oxidative phosphorylation (OXPHOS). The identified protein changes do not reflect the level of the whole protein complexes as they function in-vivo. In the present study, we investigated in detail the age-dependent changes of assembled mitochondrial protein complexes, using complexome profiling. We observed pronounced age-depen-dent alterations of the OXPHOS complexes, including the loss of mitochondrial respiratory supercomplexes (mtRSCs) and a reduction in the abundance of complex I and complex IV. Additionally, we identified a switch from the standard complex IV-dependent respiration to an alternative respiration during the aging of the *P. anserina* wild type. Interestingly, we identified proteasome components, as well as endoplasmic reticulum (ER) proteins, for which the recruitment to mitochondria appeared to be increased in the mitochondria of older cultures. Overall, our data demonstrate pronounced age-dependent alterations of the protein complexes involved in energy transduction and suggest the induction of different non-mitochondrial salvage pathways, to counteract the age-dependent mitochondrial impairments which occur during aging.

## 1. Introduction

Biological aging is commonly described as a process associated with the time-dependent decline of physiological functions and the accumulation of cellular and molecular damage [1,2,3]. Over decades of research, mitochondria have been demonstrated to play a central role in aging [4,5,6,7,8,9]. These organelles are involved in a number of essential cellular processes, including the synthesis of iron/sulfur clusters, amino acid, lipids, and in energy transduction leading to the production of ATP. A network of pathways involved in maintenance of a population of intact mitochondria is effective and counteracts the time-dependent accumulation of functionally impaired mitochondria [10,11].

One model organism with a strong mitochondrial etiology of aging is the ascomycete *Podospora anserina* [12,13,14]. This filamentous fungus is characterized by a limited lifespan. Starting from a single ascospore, the product of sexual reproduction, a vegetation body (mycelium) develops that consists of branched filamentous cells (hyphae), which grow at their tips. After a strain-specific linear growth period of a few weeks, the growth rate of the mycelium slows down. Finally, growth ceases and the hyphal tips burst [15,16]. During aging of *P. anserina* the mitochondria undergo pronounced morphological and ultrastructural changes. In juvenile hyphae, the mitochondria occur as a network of filamentous organelles. Due to an altered balance of the mitochondrial fusion and fission, the network disintegrates during aging, leading to punctuated mitochondria [17]. At the ultrastructural level, the typical lamellar cristae ultrastructure changes and forms vesicles [18,19]. This age-dependent structural reorganization is concurrent with the dissociation of F_1_F_o_-ATP synthase dimers [20]. Recently, intervention in the F_1_F_o_-ATP-synthase dimerization process, via the ablation of the F_1_F_o_-ATP-synthase assembly factor PaATPE, experimentally supported this scenario. The *PaAtpe* deletion mutant displays a vesicular mitochondrial ultrastructure and impairments in mitochondrial function and is short-lived, due to a detrimental induction of mitophagy [21,22]. Age-dependent fragmentation of mitochondria and reorganization of the inner membrane were also reported for other organisms, from yeast to humans [17,23,24,25,26,27,28].

In *P. anserina*, the age-related changes in mitochondrial morphology and ultrastructure go along with a pronounced reorganization of the mitochondrial DNA (mtDNA), which encodes a number of essential proteins of the respiratory chain [29,30,31,32,33]. Stabilization of the mtDNA by various means was shown to extend lifespan [34,35,36,37]. These data suggested an important role of mitochondrial respiration in the aging process of *P. anserina* and this was found to be evolutionary conserved [7,38,39,40]. Aging was further linked to mitochondrial impairments, such as reduction of the capacity of the oxidative phosphorylation (OXPHOS) at the respiratory chain and, thus, an age-dependent decrease of respiration [24,41,42,43,44]. A variety of studies with *P. anserina* further supported the impact of respiration on aging and lifespan. For instance, impairments of the standard complex IV (cytochrome-c-oxidase; COX)-dependent respiration in different *P. anserina* mutants led to the induction of an alternative oxidase (AOX)-dependent respiration and an increased lifespan [36,37,45,46]. This increase is concomitant with a reduction of the generation of the superoxide anion as a reactive oxygen species (ROS) [46]. Details about the role of ROS in the aging of *P. anserina* were discussed in a recent review [14]. A general role of ROS, which to a large extend are generated at the mitochondrial respiratory chain, and aging is evolutionary conserved from yeast to humans and is the kernel of the ‘mitochondrial free radical theory of aging’ [5]. Molecular impairments in respiration are known to increase oxidative stress and contribute to the aging process [14,47,48]. A detailed understanding of the structure and function of the respiratory machinery is therefore of paramount relevance for understanding biological aging. The respiratory chain consists of five individual complexes (complex I–V), which can assemble into different supercomplexes, the so-called mitochondrial respiratory supercomplexes (mtRSC) [49]. Individual OXPHOS complexes and mtRSCs exist side by side in the inner mitochondrial membrane [50,51]. In addition, in *P. anserina,* such a structure was previously demonstrated [52]. The manipulation of the composition of the respiratory chain was shown to have a strong impact on lifespan. For instance, disruption of mitochondrial respiratory supercomplex (mtRSC) assembly by ablation of PaRCF1 and PaRCF2, two mtRSC assembly factors, leads to lifespan shortening [53]. Recently, a study of the *P. anserina* F_1_F_o_-ATP synthase revealed hints at an age-dependent alteration of the respiratory chain composition and loss of mtRSCs [21]. Moreover, the pronounced lifespan-extending effect in a mutant deleted for the gene coding for the mitochondrial PaIAP protease was found to be linked to an increase in mtRSCs [54].

In the past, a number of studies focused on age-dependent changes in mitochondrial transcript and protein levels in different biological systems, including *P. anserina*. A longitudinal transcriptome analysis revealed that the transcript profiles associated with mitochondrial function fluctuate during the aging of *P. anserina* [55]. This fluctuation has also been studied on the protein level in *P. anserina,* but mainly with the soluble protein part of mitochondria and a focus on protein modifications [56,57,58]. Only one study addressed both soluble and membrane-integral mitochondrial proteins and demonstrated age-dependent changes of some individual mitochondrial proteins associated with the OXPHOS [59]. However, in none of these studies was special care taken to prepare and analyze protein complexes and, thus, they do not provide information about alterations at the level of whole protein complexes.

Here we report results from a complexome profiling analysis, in which high molecular weight protein complexes were separated using BN-PAGE, subsequently sliced into even fractions, and analyzed by quantitative mass spectrometry [60,61,62]. The identified proteins were quantified and their appearance in high molecular weight regions could be visualized as interaction profiles, using heatmaps [63]. Here, the comparison of the resulting profiles of two mitochondrial samples, from two different age stages of the *P. anserina* wild type, uncovered detailed age-dependent alterations of the mitochondrial complexome. We also observed an age-associated switch from a standard complex IV-dependent, to an AOX-dependent, respiration in the *P. anserina* wild type. Most strikingly, we identified that components of the proteasome, as well as the endoplasmic reticulum (ER) proteins, had an increased abundance of enriched mitochondrial fractions of older strains, suggesting the induction of compensatory salvage pathways occurs during aging.

## 2. Materials and Methods

### 2.1. P. anserina Strains and Cultivation

In this study, the *P. anserina* wild-type strain ‘s’ [64] was used. The strain was grown on standard cornmeal agar (BMM) at 27 °C under constant light [65]. For all analyses in this study, strains derived from monokaryotic ascospores were used [65]. These spores were isolated from defined crosses. For germination, spores were incubated on standard cornmeal agar (BMM) with 60 mM ammonium acetate (Merck, Darmstadt, Germany; 1116.1000) at 27 °C in the dark for 2 days. To obtain cultures with a defined age, a piece of germinated mycelium was placed at one site of a M2 agar plate and incubated for the desired time (i.e., 6 or 18 days) at 27 °C under constant light.

### 2.2. Isolation of Mitochondria

*P. anserina* wild-type strains were grown on cellophane foil covered M2 agar plates under constant light at 27 °C. After two days of growth, mycelia were transferred to CM-liquid medium and incubated for an additional two days at 27 °C, in constant light and shaking. The mitochondria of young (6 d) and old (18 d) *P. anserina* wild-type cultures were isolated, according to a published protocol [46]. For purification of mitochondria a discontinuous sucrose gradient (20–36–50%) and ultracentrifugation (100,000× *g*) was used [65].

### 2.3. Blue Native Gel Electrophoresis (BN-PAGE)

BN-PAGE was performed, to separate native protein complexes according to size, as described [66,67]. Briefly, 150 μg of isolated mitochondria (mitochondria from two independently isolations were pooled) were solubilized with digitonin (Sigma-Aldrich, St. Louis, MO, USA; D141) at a detergent/protein ratio of 4 g/g. Solubilized mitochondria were centrifuged at 4 °C for 30 min at 20,000× *g*. The obtained supernatants were immediately loaded on a linear gradient gel (4–13%) overlaid with 3.5% stacking gel. Electrophoresis was started at 100 V and 10 mA. When samples had completely migrated into the stacking gel, limits were set to 500 V and 15 mA. Electrophoresis was stopped when the Coomassie front approached the gel front. After electrophoresis, the gel was incubated for 30 min in fixing solution (50% methanol (Carl Roth, Karlsruhe, Germany; 4627.5), 10% acetic acid (Carl Roth, Karlsruhe, Germany; 3738.5), 100 mM ammonium acetate (Merck, Darmstadt, Germany; 1116.1000), and subsequently proteins were visualized after 1-h Coomassie staining (0.025% Coomassie blue-G 250 (Carl Roth, Karlsruhe, Germany; 9598.2) in 10% acetic acid (Carl Roth, Karlsruhe, Germany; 3738.5). Afterwards the gel was destained with water and 10% acetic acid.

### 2.4. Western Blot Analysis

Mitochondrial protein extracts (50 µg) were separated by 2-phase SDS-PAGE (12% separating gels) according to the protocol published in Brust et al. [18]. Briefly, after electrophoresis of mitochondrial protein extracts, proteins were transferred to PVDF membranes using the Trans-Blot^®^ Turbo™ transfer system (BIO-RAD, Hercules, CA, USA), according to the manufacturer’s specifications. Afterwards, blocking, antibody incubation, and washing steps were performed according to the Odyssey ‘Western Blot Analysis’ handbook (LI-COR Biosciences, Bad Homburg, Germany). As primary antibody, an anti-PaAOX1 (rabbit, 1:5000 diluted, raised against synthetic peptide NHKEDPNPFVSDYKCDADHQR, Davids Biotechnologie GmbH, Regensburg, Germany) was used. Subsequently, a conjugated IR Dye CW 800 (1:15,000 dilution, goat anti-rabbit 800: LIC-OR Biosciences, Bad Homburg, Germany; 926-32211) was used as secondary antibody. For detection, an Odyssey^®^ Fc imaging system (LI-COR Biosciences, Bad Homburg, Germany) was used, and densitometric quantification was performed with the manufacturer’s software, Image Studio 5.2.

### 2.5. Mitochondrial Oxygen Consumption

Determination of COX- and AOX-dependent oxygen consumption was performed using strains cultivated on M2 medium for 2 days and in CM liquid medium for 2 days, as described above. Small pieces of mycelium were subsequently transferred into the high-resolution respirometer (Oxygraph-2k series C and G, Oroboros Instruments, Innsbruck, Austria) and oxygen consumption was measured in liquid CM medium according to the manufacturer’s instructions. Then, 1 mM potassium cyanide (KCN; Fluka, Buchs, Switzerland; 60178) was added to inhibit respiration via COX, and 4 mM salicylhydroxamic acid (SHAM; Sigma-Aldrich, St. Louis, MO, USA; S607) was added to inhibit respiration via AOX. Data were analyzed using the manufacturer’s software DatLab 6.

### 2.6. Complexome Profiling

Complexome profiling was used to identify native protein complexes after BN-PAGE and their alterations during aging. It was performed as published previously [61]. In brief, 150 µg isolated mitochondria (mitochondria from two independent isolations were pooled), solubilized, and separated by BN-PAGE, as described above in the BN-PAGE section. After native electrophoresis, blue-native gels were fixed in 50% (*v*/*v*) methanol (Thermo Fisher Scientific, Waltham, MA, USA; 11976961), 10% (*v*/*v*) acetic acid (Merck, Darmstadt, Germany; 33209), and 10 mM ammonium acetate (Merck, Darmstadt, Germany; 1116.1000) for 30 min and stained with Coomassie (0.025% Serva Blue G (SERVA, Heidelberg, Germany, 35050) and 10% (*v*/*v*) acetic acid (Merck, Darmstadt, Germany; 33209)). Afterwards, each lane of the gel was cut from the bottom to the top into 48 almost equal fractions. Fractions were collected in 96 filter well plates (30–40 µm PP/PE, Pall Corporation). The gel pieces were destained in 60% Methanol, 50 mM ammonium bicarbonate (ABC) (Merck, Darmstadt, Germany; V900254). Solutions were removed by centrifugation for 2 min at 600× *g*. Proteins were reduced in 10 mM DTT (Merck, Darmstadt, Germany; D9779) and 50 mM ABC (Merck, Darmstadt, Germany; V900254) for one hour at 56 °C and alkylated for 45 min in 30 mM iodoacetamid (Merck, Darmstadt, Germany; I1149). Samples were digested for 16 h with trypsin (Promega, Fitchburg, WI, USA; V5111), at 37 °C in 50 mM ABC (Merck Darmstadt, Germany; V900254), 0.01% Protease Max (Promega, Fitchburg, WI, USA; V2072), and 1 mM CaCl_2_ (Merck, Darmstadt, Germany; C3306). Peptides were eluted in 30% acetonitrile (VWR, Radnor, PA, USA; 83640) and 3% formic acid (VWR, Radnor, PA, USA; 85048), centrifuged into a fresh 96 well plate, dried in a speed vac, and resolved in 1% acetonitrile and 0.5% formic acid.

Liquid chromatography/mass spectrometry (LC/MS) was performed on a Thermo Scientific™ Q Exactive Plus equipped with an ultra-high-performance liquid chromatography unit (Thermo Scientific Dionex Ultimate 3000) and a Nanospray Flex Ion-Source (Thermo Scientific). Peptides were loaded on a C18 reversed-phase precolumn (Thermo Scientific), followed by separation with a 2.4 µm Reprosil C18 resin (Dr. Maisch GmbH) in-house packed picotip emitter tip (diameter 100 µm, 15 cm from New Objectives), separated using a gradient from 4% acetonitrile, 0.1% formic acid, to 30% eluent B (99% acetonitrile, 0.1% formic acid) for 30 min and an additional gradient to 60% for 5 min with a flow rate 400 nL/min and washout with 99% B for 5 min. MS data were recorded by data-dependent acquisition. The full MS scan range was 300 to 2000 *m*/*z*, with a resolution of 70,000 and an automatic gain control (AGC) value of 3E6 total ion counts, with a maximal ion injection time of 160 ms. Only higher charged ions (2+) were selected for MS/MS scans with a resolution of 17,500, an isolation window of 2 *m*/*z*, and an automatic gain control value set to E5 ions, with a maximal ion injection time of 150 ms. MS1 data were acquired in profile mode.

MS data were analyzed using MaxQuant (v 2.0.1.0) [68] and the default settings. Proteins were identified using the *P. anserina* proteome database UniProtKB with 10657 entries, released in 8/2021. The enzyme specificity was set to trypsin. Acetylation (+42.01) at the *n*-terminus and oxidation of methionine (+15.99) were selected as variable modifications, and carbamidomethylation (+57.02) as fixed modification on cysteines. The false discovery rate (FDR) for the identification protein and peptides was 1%. Intensity-based absolute quantification (IBAQ) values were recorded. The sum of all IBAQ values of data sets were normalized to data of the 18-day-old sample. Protein abundance within native lanes were normalized to the maximum appearance, to enable comparison of mitochondrial complexes between samples. Slice number, according to a native mass ladder, was used for native mass calibration. The software NOVA (v.0.5.7) was used for hierarchical clustering of complexomics data [69].

## 3. Results and Discussion

### 3.1. Aging in P. anserina Leads to Changes in the Composition of the Respiratory Chain

The pronounced age-associated reorganization of the mtDNA of *P. anserina,* resulting in the deletion of large parts of the mtDNA, suggested that severe changes of the mitochondrial transcriptome and proteome occur during aging [29,30,31,32,33]. Subsequent studies verified this assumption [55,56,57,58,59]. For instance, the transcript and protein level of mitochondrial-encoded OXPHOS subunits such as PaCOX1 (Pa_mito_cox1), PaCOX2 (Pa_mito_cox2), and PaND4 (Pa_mito_nad4) clearly decline during aging. Obviously, such alterations impinge on the abundance of the individual OXPHOS complexes and subsequently on the level of the mtRSCs. Accordingly, in older cultures a decreased abundance of mtRSCs compared to young cultures was found [21].

**Figure 1:**
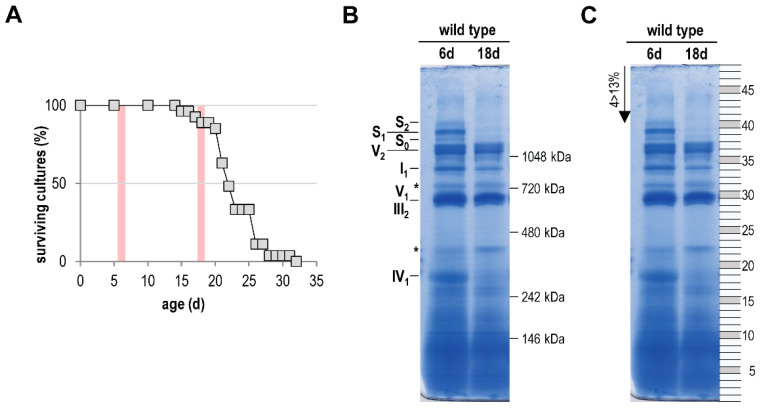
Age-dependent reduction of respiratory chain complexes. (**A**) Survival curve of *P. anserina* wild type (*n* = 15) grown on M2 medium under standard growth conditions. Red bars mark the time points used for further investigations. (**B**) BN-PAGE analysis of isolated mitochondria from 6-day- and 18-day-old wild-type cultures (each sample was pooled from the mitochondria of two different cultures). Mitochondria were solubilized with digitonin (ratio: digitonin to protein 4 g/g). The CI_1_CIII_2_CIV_0–2_ (S_0–2_) supercomplexes, dimeric complexes III and V (III_2_ and V_2_), as well as monomeric complexes I_1_, IV_1_, and V_1_ were visualized using Coomassie staining. Asterisks (*) mark uncharacterized protein bands. (**C**) For complexome profiling the BN-PAGE lanes were cut horizontally from the lower to the upper site in 48, almost equally sized, pieces. The numbers of the single fractions are indicated on the right.

In the current study, we aimed to focus on a comprehensive analysis of the age-related mitochondrial complexome [60,61,62]. For this analysis we isolated mitochondria from 6-day- and 18-day-old *P. anserina* wild-type cultures. The older age-stage lies in the range were the first cultures of a population died (Figure 1A). After solubilization with the mild detergent digitonin, the mitochondrial proteins (each sample was pooled from mitochondria of two different cultures) were separated by BN-PAGE. In the case of the 6-day-old sample, we obtained the typical and well-known pattern of OXPHOS complexes [52,70]. Coomassie staining visualizes complex I (NADH dehydrogenase), complex III (cytochrome c reductase), and complex IV (cytochrome oxidase; COX), as well as monomeric and dimeric complex V (F_1_F_o_-ATP-synthase). Furthermore, all three known mtRSCs, S_0_, S_1_, and S_2_, which consist of CI_1_CIII_2_CIV_0_, CI_1_CIII_2_CIV_1_, and CI_1_CIII_2_CIV_2_, respectively, were visible (Figure 1B). In agreement with a previous study [21] mtRSCs were strongly reduced or partly lost in the 18-day-old sample. A comparable alteration of mtRSCs was also found in the mitochondria of aged rat cells [71,72]. Furthermore, a reduction of monomeric complex I (I_1_) and monomeric complex IV (IV_1_) occurred, suggesting a reduced activity of these complexes in aged cultures. Such a reduced activity of complex I and complex IV was found in aged rodents [73,74,75].

Next, 48 equal pieces (Figure 1C) of the BN-PAGE from each of the two samples were fractionated and subjected to a quantitative mass spectrometry analysis. Identified proteins were clustered according to their abundance profile across all fractions (Table S1A), and a soluble mass ladder (Figure 1B, Table S1B) was used for native mass calibration of complexome data (Table S1B). Overall, 1314 unique proteins were identified (Table S1A). Many of the clustered proteins form parts of well-studied protein complexes, such as the five protein complexes of the OXPHOS system. We focused our attention on the OXPHOS complexes and on other yet unstudied major complexes with an altered abundance in the two mitochondrial samples from cultures of different age.

**Figure 2:**
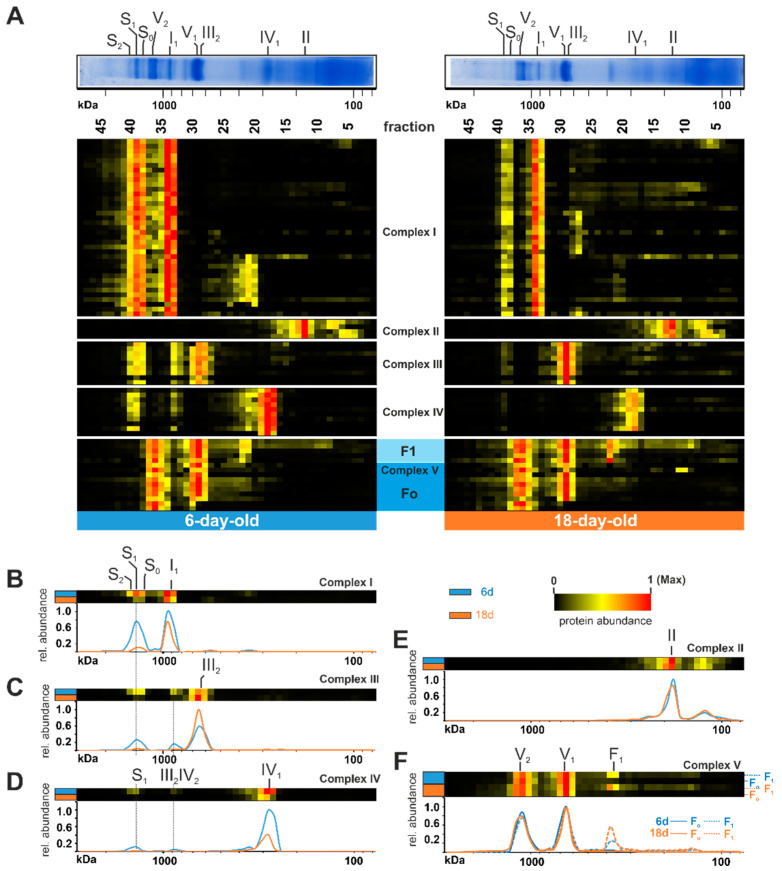
Age-dependent changes of respiratory chain complexes. (**A**) Identified subunits of all complexes of the oxidative phosphorylation system are represented in a heat map. The relative abundances were normalized to the maximum value within each lane. The color scale ranges from black (not identified), to yellow (20% of the maximum in (**A**), 15% in (**B**–**F**)), to red (maximum abundance). For detailed information about the corresponding proteins refer to Table S1C. Heatmaps of a combination of all respiratory chain complexes and visualization in 2D profiles for (**B**) complex I, (**C**) complex III (**D**) complex IV, and (**E**) complex II. (**F**) Complex V (ATP synthase) with the appearance of F_1_ and F_o_ domains.

To reveal age-dependent changes in the OXPHOS, we generated a heat map and compared the distribution profiles of both age stages (Figure 2). An age-related reduction of mtRSCs is clearly seen in the decreased abundance of proteins belonging to complex I, complex III, and complex IV in fractions 38 to 40 (mass range 1200 kDa up to 1600 kDa) (Figure 2A). This is in good agreement with previously published data obtained in an analysis about the role of F_1_F_o_-ATP-synthase dimers in the aging of *P. anserina* [21]. Such an age-dependent reduction of mtRSCs was also observed in different rat tissues [71,72]. In cardiac mitochondria from aged rats, it was demonstrated that the mtRSCs with the highest molecular masses were diminished [72]. Additionally, in mitochondria from the brain cortex of aged rats, a loss of mtRSCs stability was described [71]. Furthermore, mtRSCs play a role in various human diseases, such as Barth syndrome, heart failure, and Parkinson’s disease [76,77,78,79]. In addition to changes in mtRSC abundance, we observed changes in the individual complexes of the OXPHOS. Particularly, the abundance of complex I subunits was reduced from 6-day-old to 18-day-old samples in fractions 33 and 34 (position of individual complex I at ~900 kDa) (Figure 2A,B). A more detailed evaluation of these changes follows in a subsequent section. The total abundance of complex III did not change (Table S1C). Instead, during aging, complex III containing mtRSCs disintegrated into individual complex III dimers (Figure 2A,C). Similarly to complex I, the amount of complex IV was reduced in older cultures (Figure 2D). The age-dependent dissociation of the complex IV-containing mtRSCs should instead increase the amount of complex IV monomer. However, the amount of monomeric complex IV was much lower, suggesting additional reasons for the loss of this complex and supercomplexes (Figure 2D). Minor alterations were observed in complex II (Figure 2A,E). Here, the total amount of individual subunits was not affected. In contrast to the other complexes, complex V exists in a monomeric (V_1_), as well as in a dimeric (V_2_), state [70]. An age-related dissociation of complex V dimers, as described in earlier studies [20,21], was not observed in the current analysis (Figure 2A,F). However, due to the use of different cultivation media, the cultures in the previous study were physiologically older than in the current study, suggesting that the dissociation of complex V dimers may occur at the end of life. Interestingly, some subunits of complex V were found in lower fractions with an increased abundance in the older sample (lower panel of Figure 2A). These subunits are part of the F_1_ domain of complex V, indicating an imbalanced assembly of F_1_ and F_o_ domains (Figure 2F).

Overall, these data identified clear age-dependent alterations of OXPHOS in *P. anserina,* which ultimately must lead to impaired mitochondrial function.

#### 3.1.1. The Alternative NADH Dehydrogenases PaNDI1 Counterbalances Complex I Deficiency during Aging

**Figure 3:**
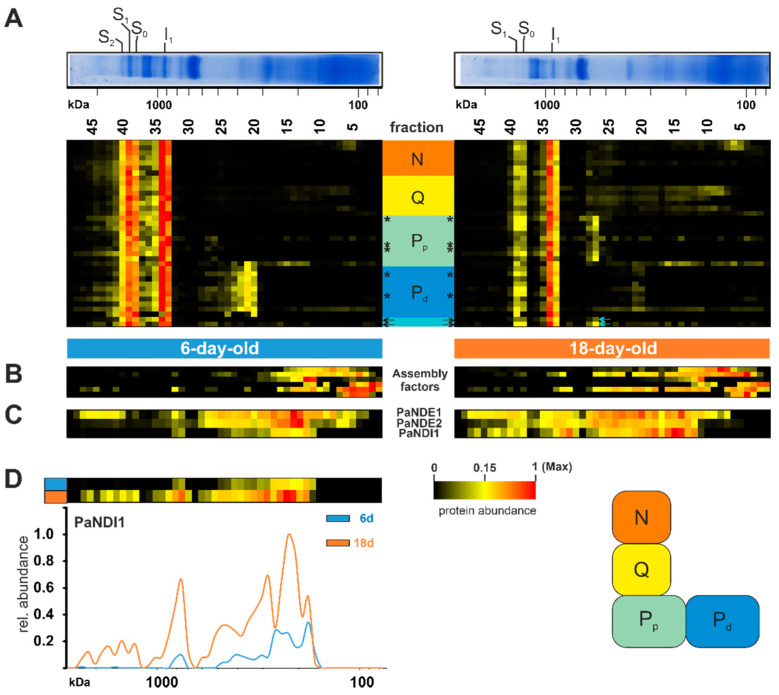
Increase of alternative NADH dehydrogenase PaNDI1 during aging. (**A**) Quantified proteins of complex I, assignment to modules, complex I assembly factors, (**B**) homologs of complex I assembly factors and (**C**) the alternative NADH dehydrogenases PaNDI1 (Pa_7_1820), PaNDE1 (Pa_1_24200) and PaNDE2 (Pa_7_5390) are represented in heat maps. Mitochondrial-encoded proteins are marked by asterisks (*). Complex I modules were assigned according to [80] N-module (N) in orange, Q-module (Q) in yellow, proximal proton pumping module (Pp) in light green, distal proton pumping module (Pd) in blue. Two unassigned subunits marked in light cyan. The color scale of heatmaps ranges from black (not identified), to yellow (15% of the maximum), to red (maximum abundance). (**D**) Distribution profiles of PaNDI1 in 6-day-old (grey line) and 18-day-old (orange line) wild-type mitochondria. For detailed information about the corresponding proteins refer to Table S1D.

Next, we investigated complex I in more detail (Figure 3A). This complex consists of several subunits with seven mitochondrial- and more than 34 nuclear-encoded; although the complete list of all complex I subunits is still not known. In the complexome dataset, a total of 37 subunits, with five being mitochondrial-encoded, were detected (*, Figure 3A). In total, we observed a reduced amount of complex I in the older *P. anserina* wild-type cultures (Figure 2B and Figure 3A, Table S1D). Noticeably, several complex I subunits are rarely found in fractions under 500 kDA (fraction 25 and lower). Different studies demonstrated a modular structure and stepwise assembly of complex I via different intermediates [60,80,81,82,83]. Intermediates were identified and more closely characterized in complexome profiling of *Arabidopsis thaliana* [84] and human cell cultures [81]. Subunits of the structural modules (Figure 3) were sorted according to their homologs in mammals and *Yarrowia lipolytica,* and listed in Table S1D [85]. In contrast to complexome profiling data from mammals, *P. anserina* exhibited an accumulation of distal membrane arm domains, with a size of 300–500 kDa, in the 6-day-old sample. It remains unclear if this subcomplex is a preassembly or the breakdown product of a complex I disassembly during maintenance. The latter was recently suggested in mammalian mitochondria, where a maintenance pathway for complex I was described [86,87].

Interestingly, this membrane arm subcomplex was not accumulated in the 18-day-old samples, indicating that this module is not in stock and assembly or maintenance was delayed. On the other hand, the 18-day-old sample accumulated the proximal part of the proton pumping module (Figure 3A). Little is known about assembly factors in *P. anserina*. None of the identified homologs to human assembly factors had clearly migrated together with the accumulated membrane arm intermediates (Figure 3B, Table S1D). Two subunits (Pa_1_16630 and Pa_2_5150) could not be assigned to any known homolog of complex I subunits. Both proteins shared a migration through the native gel with subunits assigned to the proximal proton pumping module, suggesting their structural location (Figure 3A, arrows).

Under standard growth conditions, complex I-dependent NADH oxidation is coupled with proton-pumping. In plants and fungi, this oxidation can also occur through another family of proteins. These so-called alternative NADH dehydrogenases oxidize NADH from the cytosol (external enzymes) or from the mitochondrial matrix (internal enzymes) without proton pumping [88]. In the yeast *Saccharomyces cerevisiae,* which lacks complex I, three alternative NADH dehydrogenases, one internal and two external enzymes, have been well characterized [89,90]. However, some organisms with complex I also possess alternative NADH dehydrogenases. In *P. anserina* the three alternative NADH dehydrogenases, PaNDI1 (internal, Pa_7_1820), PaNDE1 (external, Pa_1_24200), and PaNDE2 (external, Pa_7_5390), are active [46,91]. A previous study showed that PaNDI1 acts as a molecular bypass to restore the physiological consequences of complex I deficiency in *P. anserina* mutants. Interestingly, both external NADH dehydrogenases are not able to compensate complex I deficiency in *P. anserina* [91]. Furthermore, expression of NDI1 from *S. cerevisiae* partially restores complex I deficiencies in other organisms, such as worms and mice, and in human cell lines [92,93,94,95,96]. Whether the induction of alternative NADH dehydrogenases plays a role during aging has not been analyzed so far. Our current data demonstrate an age-dependent reduction of both external NADH dehydrogenases PaNDE1 and PaNDE2 (Figure 3C), whereas the internal NADH dehydrogenases PaNDI1 showed an increased abundance in the older sample (Figure 3C,D). We assume that PaNDI1 is induced as a compensatory mechanism, to overcome the age-associated reduction of complex I, and prevents an over-reduction of the NAD^+^/NADH pool in the mitochondrial matrix. Altogether, this is the first hint at a role of PaNDI1 in the aging process of *P. anserina*.

#### 3.1.2. Age-Dependent Induction of Alternative Respiration via PaAOX

**Figure 4:**
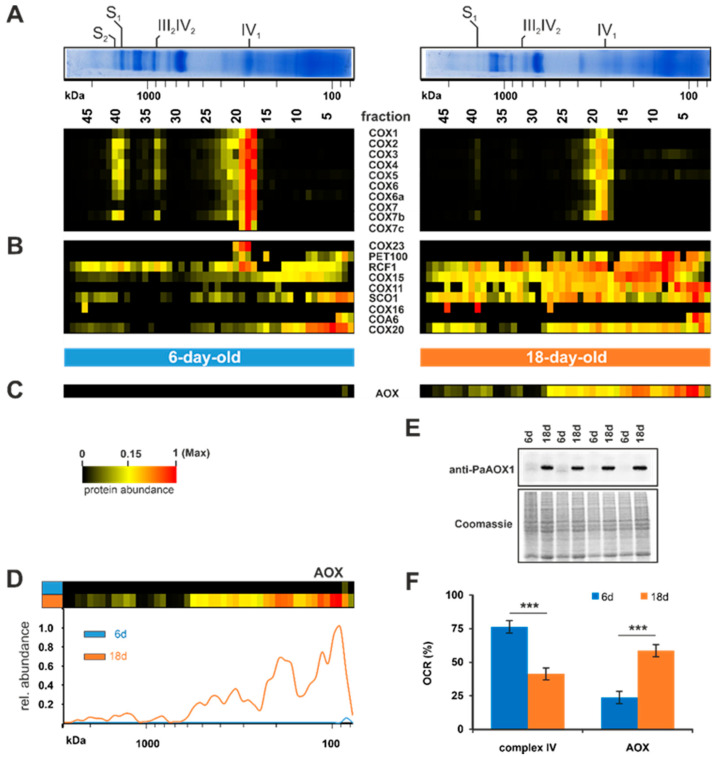
Age-dependent switch to AOX-dependent respiration. (**A**) Quantified subunits of complexes IV, (**B**) additional complex IV proteins and assembly factors, and (**C**) the alternative oxidase (AOX) are represented in a heat map. The color scale ranges from black (not identified), to yellow (15% of the maximum), to red (maximum abundance). (**D**) Complexome distribution profiles of alternative oxidase of 6-day-old and 18-day-old wild-type cultures. (**E**) Western blot analysis of mitochondrial protein extract of 6-day- and 18-day-old *P. anserina* wild-type cultures (each with 4 biological replicates). A specific PaAOX1 antibody was used to detect the ~34 kDa alternative oxidase (PaAOX). Coomassie staining served as loading control. (**F**) Relative complex IV- and AOX-dependent oxygen consumption rate (OCR) of 6-day- and 18-day-old wild-type mycelium after treatment with SHAM (AOX-inhibitor) or KCN (complex IV-inhibitor).

In the next set of analyses, we investigated the reduction of complex IV in more detail. Complexome profiling identified ten subunits of complex IV, whose abundance was reduced during aging (Figure 4A). Among these are the three mitochondrial-encoded catalytic subunits, PaCOX1, PaCOX2, and PaCOX3. PaCOX1 was one of the most reduced subunits in the 18-day-old sample compared to the 6-day-old sample (upper lane in Figure 4A). Since COX1 initiates the complex IV assembly [97,98], it is possible that this strong reduction affects the other subunits and leads to their reduction in quantity. Furthermore, the huge reduction of this mitochondrial-encoded subunit was expected, due to the age-related reorganization of the mtDNA and the reduction of the number of mtDNA copies [29,30,31,32,33]. Another explanation for the reduced PaCOX1 amount could be the fact that an intron of the *PaCox1* gene codes for the plDNA, which becomes liberated during aging, leading to the mtDNA reorganization [30,31,32]. To reduce excessive plDNA formation, transcription of the *PaCoxI* gene might be downregulated. Indeed, a reduced *Pa Cox1* transcript level was found in a previous transcriptome analysis [55]. Other associated factors such as RCF1 and complex IV assembly factors were elevated in old mitochondria, indicating stalled assembly (Figure 4B).

In *P. anserina*, the standard complex IV-dependent respiration is known to be bypassed under certain conditions by respiration via an alternative oxidase (AOX), e.g., under the oxidative stress induced by paraquat [99]. Another example of AOX induction is the loss of complex IV, which in the absence of an alternative respiration is lethal. The *P. anserina* mutant strains grisea, *PaCox17*::*ble,* and *PaCox5*::*ble* all are devoid of intact complex IV and respire via the AOX. In these mutants AOX induction leads to lifespan extension [37,45,46,52,100,101]. Whether the induction of the alternative respiration plays a role in the wild type of *P. anserina* has hardly been analyzed. An initial study analyzing PaAOX in the aged *P. anserina* wild type revealed that induction of alternative respiration does not occur during wild-type aging [100]. In contrast, a later genome-wide longitudinal transcriptome analysis revealed an increase of the *PaAox* transcript in middle-aged cultures, suggesting that the amount of AOX in aged cultures may also be increased [55]. Both studies used different cultivation media, leading to differences in physiological aging. Borghouts and colleagues [100] analyzed much younger cultures than Philipp and colleagues [55] and those in this study. In accordance with the transcriptome data, our current complexome dataset identified an increase of the protein amount in the 18-day-old sample compared to the 6-day-old sample (Figure 4C). We detected an increase in different fractions, with the strongest increase in fraction 3 and 4, suggesting that PaAOX acts as a monomeric state (~34 kDa), but also as a dimer or an oligomer (Figure 4D). This possibility is supported by an earlier study, where two signals were detected in a western blot analysis of a 2D-BN-PAGE [52]. Consistently, a dimeric form of the AOX was reported in other fungi and plants [102,103,104] and in oligomeric forms in mice expressing *Ciona intestinalis* AOX [105].

To verify the PaAOX quantity, we conducted a western blot analysis of mitochondrial extracts from both age states (Figure 4E). This analysis confirmed the increase of PaAOX in 18-day-old cultures. In 6-day-old cultures PaAOX was hardly detectable, whereas in the older cultures a huge amount was observed. To test whether this increase affects respiration, we performed oxygen consumption measurements with intact mycelia of 6-day-old and 18-day-old cultures (Figure 4F). Using the specific inhibitors potassium cyanide (KCN) for complex IV and salicylhydroxamic acid (SHAM) for AOX, we discriminated between standard respiration (complex IV-dependent) and alternative respiration (AOX-dependent). Interestingly, the higher amount of protein PaAOX detected indicates a shift of the respiration type. Consistent with earlier published data [46], young *P. anserina* wild-type cultures (6-days-old) respire predominantly (75%) via complex IV, and only moderately (25%) via AOX. In older cultures, this is almost turned around. We determined only 40% complex IV-dependent respiration and 60% alternative respiration (Figure 4F). The increase of alternative respiration was not as high as the increase of the PaAOX amount. This is possibly due to the fact that alternative oxidases are post-translationally regulated. In different studies with plants, it was shown that gene expression and protein formation do not determine the AOX activity. Rather, the AOX activity is regulated via the oxidation state of cysteine residues between the monomers [106]. These results clearly demonstrate that during the aging of *P. anserina* wild type alternative respiration is induced. AOX-dependent respiration bypasses complex III, which is a major contributor to mitochondrial superoxide anion production [46]. In various organisms, reactive oxygen species (ROS), such as superoxide anion, have been demonstrated to induce AOX-dependent respiration [99,107,108,109,110]. We therefore assume the well-described age-dependent accumulation of ROS in *P. anserina* [17,99,111] is responsible for AOX induction. Ultimately, this adaptive process is aimed at reducing the mitochondrial ROS load. Accordingly, in the *P. anserina* grisea mutant, a long-lived mutant with AOX-dependent respiration, a striking decrease in superoxide anion production was observed [46,112,113].

Taken together, we show that alternative respiration via PaAOX plays a role in the aging process of the *P. anserina* wild type.

### 3.2. Mitochondrial Translation Machinery Declines during Aging

**Figure 5:**
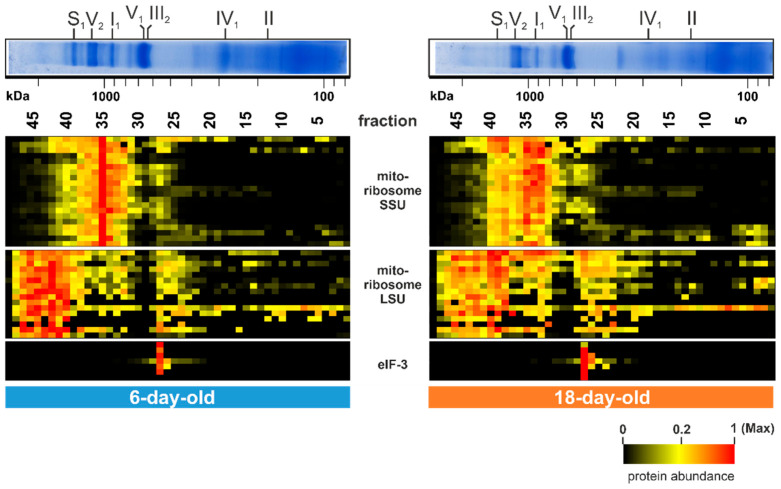
Translation of mitochondrial proteins alters during aging. Quantified proteins of the mitochondrial ribosome (large subunit; LSU and small subunit; SSU) and the translation initiation factor 3 (eIF-3) are represented in a heat map. The color scale ranges from black (not identified), to yellow (20% of the maximum), to red (maximum abundance). For detailed information about the corresponding proteins refer to Table S1E.

Beside the described age-dependent alterations further noticeable changes were detected in gel fraction 35 (1 MDa) and in fractions between 40 (1.5 MDa) and 46 at 2.4 MDa (Figure 5). The 20 corresponding proteins in fraction 35 are part of the small subunit (SSU) of the mitochondrial ribosome and the 16 clustered proteins between fractions 40 and 46 are part of the large subunit (LSU) of the mitochondrial ribosome. The SSU as well as the LSU amount decline during aging and were partly found to be disintegrated in the 18-day-old sample (Figure 5). This suggests a breakdown of the mitochondrial ribosome during aging of the *P. anserina* wild type. An age-associated change of the translation machinery was also found in various other organisms [114]. This decline of fully assembled mitochondrial ribosomes, together with the well-described decrease in mtDNA copy number [29,30,31,32,33], ultimately explains the dramatic decrease in the level of mitochondrial encoded proteins.

A further striking alteration is observed in fraction 27 (550 kDa). The seven corresponding proteins were more abundant in the 18-day-old sample than in the 6-day-old sample (lower panel of Figure 5). All of these proteins are part of the translation initiation factor 3 (eIF-3). In the filamentous fungus *Neurospora crassa*, which is closely related to *P. anserina*, but also in humans, the eIF-3 consists of 13 subunits, eight of them form the core structure [115,116,117]. The seven identified proteins are homologs of these core subunits. Age-dependent alterations of these proteins suggest an age-dependent increase of the mitochondrial association of this complex. Since, from other organism this factor is known to be part of the translation machinery of nuclear-encoded OXPHOS proteins, mitochondrial transmembrane proteins, and of the citrate cycle [118], its increased, mitochondrial association might reflect an imbalance in the co-translation of mitochondrial and nuclear-encoded OXPHOS subunits.

Overall, we identified an age-dependent alteration of components involved in mitochondrial protein translation, which may have contributed to the observed decline of complexes. We assume that this alteration leads to an imbalance between mitochondrial-and nuclear-encoded proteins, inducing mitochondrial stress.

### 3.3. Non-Mitochondrial Salvage Pathways Seem to Be Induced during Aging

**Figure 6:**
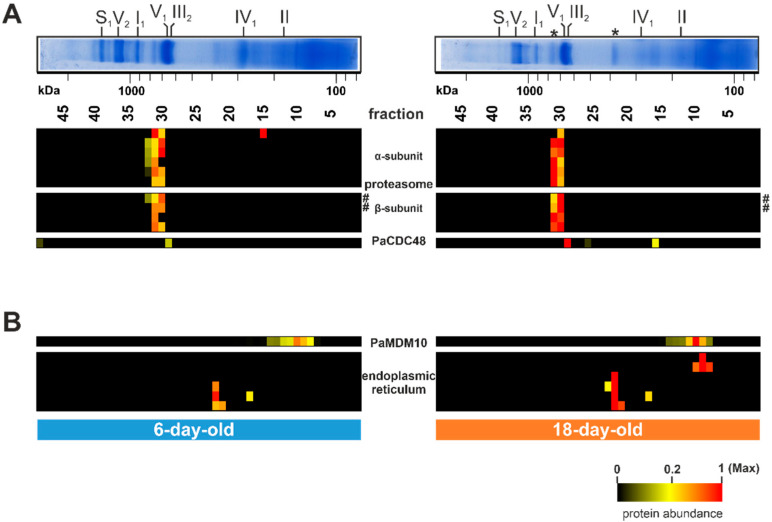
Age-dependent increase of non-mitochondrial complexes. (**A**) Quantified proteins of the proteasome and PaCDC48 (Pa_7_5590) are represented in a heat map. (**B**) Selected quantified proteins of the endoplasmic reticulum and PaMDM10 (Pa_7_11600) are represented in a heat map. The color scale ranges from black (not identified), to yellow (20% of the maximum), to red (maximum abundance). Asterisks (*) mark hitherto uncharacterized protein bands. Hashtags (#) mark two catalytic subunits (PaPRE3 and PaPRE2) of the proteasome β-subunits. For detailed information about the corresponding proteins refer to Table S1F.

Hitherto, not all distinct bands of the Coomassie-stained BN-PAGE with *P. anserina* mitochondria were attributed to defined protein complexes. Only the bands corresponding to the OXPHOS complexes have been well characterized [52,70]. However, there are still several prominent bands, such as those above complex IV or between complex V and complex I, which were not assigned to specific proteins/complexes (asterisks in Figure 1B, Figure 6A). The complexome dataset allowed us to identify the corresponding complexes. Surprisingly, these proteins are not part of known mitochondrial complexes. Instead, the identified proteins are part of cytosolic protein complexes, as well as of the endoplasmic reticulum (Figure 6A,B). Proteins of the fraction 31/32 are subunits of the proteasome and were more abundant in the older sample than in the young sample (Figure 6A; Table S1G). In yeast and mammals, the proteasome contains four rings of seven different α- and β-subunits, with molecular weights between 20 and 30 kDa. Altogether they form a complex with a total mass of about 700 kDa [119]. The complex we found has a comparable size. A total of ten proteasomal proteins were detected in our study, six from the α-subunit and four from the β-subunit (Figure 6A). The proteolytic active sites are in the inner rings of the β-subunits. Three of the seven β-subunits are proteolytic active, namely the β_1_, β_2_, and β_5_ subunits [120]. With PaPRE3 (β_1_) and PaPRE2 (β_5_) we detected two proteolytic active subunits, which were more abundant in the 18-day-old sample than in the 6-day-old sample (hashtag in Figure 6A).

The increased abundance of the proteasomal proteins suggests an increased recruitment of the proteasome to the mitochondria in the aged *P. anserina* wild type. The proteasome is an important component of the protein quality control machinery [121,122,123]. It does not only degrade cytosolic proteins, but also proteins of other cellular compartments/organelles. The degradation of damaged mitochondrial proteins via the proteasome is called ‘mitochondria-associated degradation’ (MAD) and was first discovered in *S. cerevisiae* [124]. The data presented here are the first indication of an enhanced degradation of damaged mitochondrial proteins via MAD during the aging process of *P. anserina*. A further hint at such an age-dependent MAD increase is the detection of PaCDC48, which was found in a higher amount in the older (18-day-old) compared to the young (6-day-old) sample (lower panel of Figure 6A). PaCDC48 is a homolog of the AAA-ATPase CDC48 of *S. cerevisiae* [124,125]. It is part of a protein complex binding damaged proteins and translocating them for degradation to the proteasome [126]. The increased association of the proteasome with mitochondria fits the accumulation eIF-3 in the mitochondrial fraction of aged mitochondria. To allow the formation of fully assembled OXPHOS complexes, the translation and importing of nuclear-encoded subunits needs to be well coordinated with the translation of mitochondria-encoded subunits. An imbalance of individual proteins due to reduced generation of mitochondria-encoded subunits may result in an import block of nuclear-encoded proteins. The accumulation of these proteins at the mitochondrial surface might attract the proteasome and lead to PaCDC48 recruitment as a salvage pathway.

Another possible non-mitochondrial salvage pathway may be the attachment of the ER to the mitochondrion. Indeed, we found four ER proteins in fraction 22 as part of the other prominent uncharacterized BN-PAGE band (Figure 6B; Table S1). Additional two ER-linked proteins were detected in fraction 9. All of these proteins are ER membrane proteins, suggesting that the presence of these proteins is indicative of mitochondria-ER membrane contacts. The level of these proteins increases during aging (Figure 6B) and, therefore, we suggest an increase in mitochondria-ER connections during aging. Mitochondria and the ER are linked via the ERMES (ER-mitochondria encounter structure) complex [127]. A central protein of this connecting complex is MDM10, a mitochondrial outer membrane protein [128]. In our analysis we observed an age-dependent co-migration of PaMDM10 together with ER membrane proteins in a ~150 kDa complex in fraction 10 (upper panel of Figure 6B). Intimate connections between ER and mitochondria membranes facilitate the exchange of ions, proteins, and lipids, thereby positively affecting mitochondrial function [129,130,131]. For instance, it has been demonstrated that mitochondria and ER cooperate, with the help of the proteasome, in the quality control of tail-anchored proteins [132]. Moreover, ER safeguards the import of mitochondrial proteins [132,133]. An age-dependent increase of ER–mitochondria association has also been observed in porcine aortic endothelial cells [134], suggesting that ER recruitment represents an evolutionary conserved salvage pathway. Beside the role of the ER and mitochondria in salvage pathways, these contacts may be linked to increased mitochondrial fission, as was shown to occur during the aging of *P. anserina* [17]. Such a role has been demonstrated in human cell lines, where the ER-mitochondria contact sites define the position at which the fission machinery assembles at the mitochondria before fission [135,136,137].

## 4. Conclusions

Overall, during the aging of *P. anserina,* pronounced changes in the mitochondrial complexome profile occur. The fundamental age-dependent reorganization of the respiratory chain via the induction of AOX, together with the decline in the amount of mtRSC, clearly affect the ability of the mitochondria to generate ATP. Moreover, the loss of mitochondrial ribosome subunits impairs mitochondrial translation; thereby, further compromising mitochondrial function. Interestingly and hitherto unknown, in *P. anserina* these alterations appear to induce the recruitment of components of compensatory pathways, such as the proteasome and the ER to mitochondria, to counteract these impairments. Future studies need to address the regulation of these salvage pathways in more detail, to provide a better understanding of their role in the prevention of age-dependent impairments.

## Data Availability

The complexome profiling data are available in ProteomeXchange (http://www.proteomexchange.org/) via the PRIDE [138] partner repository with the identifiers PXD029352.

## Supplementary Materials

The following are available online at https://www.mdpi.com/article/10.3390/cells10123319/s1, Table S1: Total complexome heat map.
